# Cost-Effectiveness of Serum Galactomannan Surveillance during Mould-Active Antifungal Prophylaxis

**DOI:** 10.3390/jof7060417

**Published:** 2021-05-26

**Authors:** Ai Leng Khoo, Ying Jiao Zhao, Glorijoy Shi En Tan, Monica Teng, Jenny Yap, Paul Anantharajah Tambyah, Chin Hin Ng, Boon Peng Lim, Louis Yi Ann Chai

**Affiliations:** 1 Pharmacy and Therapeutics Office, Group Health Informatics, National Healthcare Group, Singapore 138543, Singapore; ai_leng_khoo@nhg.com.sg (A.L.K.); ying_jiao_zhao@nhg.com.sg (Y.J.Z.); monica_teng@nhg.com.sg (M.T.); boon_peng_lim@nhg.com.sg (B.P.L.); 2 Department of Infectious Diseases, Tan Tock Seng Hospital, National Centre for Infectious Diseases, Singapore 308442, Singapore; glorijoy_se_tan@ttsh.com.sg; 3 Division of Infectious Diseases, University Medicine Cluster, National University Health System, Singapore 117549, Singapore; mdcpat@nus.edu.sg; 4 Department of Pharmacy, National University Health System, Singapore 117549, Singapore; jenny_yap@nuhs.edu.sg; 5 Department of Medicine, Yong Loo Lin School of Medicine, National University of Singapore, Singapore 117572, Singapore; chinhin.ng@cfch.com.sg; 6 Infectious Diseases Translational Research Programme, Department of Medicine, Yong Loo Lin School of Medicine, National University of Singapore, Singapore 117572, Singapore; 7 National University Cancer Institute, National University Health System, Singapore 117549, Singapore

**Keywords:** aspergillosis, biomarker, immunocompromised hosts, invasive fungal disease, pharmacoeconomics

## Abstract

Serial galactomannan (GM) monitoring can aid the diagnosis of invasive aspergillosis (IA) and optimise treatment decisions. However, widespread adoption of mould-active prophylaxis has reduced the incidence of IA and challenged its use. We evaluated the cost-effectiveness of prophylaxis-biomarker strategies. A Markov model simulating high-risk patients undergoing routine GM surveillance with mould-active versus non-mould-active prophylaxis was constructed. The incremental cost for each additional quality-adjusted life-year (QALY) gained over a lifetime horizon was calculated. In 40- and 60-year-old patients receiving mould-active prophylaxis coupled with routine GM surveillance, the total cost accrued was the lowest at SGD 11,227 (USD 8255) and SGD 9234 (USD 6790), respectively, along with higher QALYs gained (5.3272 and 1.1693). This strategy, being less costly and more effective, dominated mould-active prophylaxis with no GM monitoring or GM surveillance during non-mould-active prophylaxis. The prescription of empiric antifungal treatment was influential in the cost-effectiveness. When the GM test sensitivity was reduced from 80% to 30%, as might be anticipated with the use of mould-active prophylactic agents, the conclusion remained unchanged. The likelihood of GM surveillance with concurrent mould-active prophylaxis being cost-effective was 77%. Routine GM surveillance remained cost-effective during mould-active prophylaxis despite lower IA breakthroughs. Cost-saving from reduced empirical antifungal treatment was an important contributing factor.

## 1. Introduction

Invasive aspergillosis (IA) is a life-threatening infection, in patients with haematological malignancies or undergoing stem cell transplantation, with incidence ranging between 5 and 15% and mortality rates of up to 40% [1,2,3,4]. Early diagnosis is pivotal, but this is challenged by atypical presentation in immunocompromised patients. The diagnosis of IA has to be considered, taking into account: (i) host risk factors; (ii) clinical criteria including radiographic computerised tomography (CT) scans; and (iii) supporting mycology returns incorporating histopathology, culture, or diagnostic biomarkers [5]. Procedure-based histopathologic detection of the fungus or culture from biopsy specimens is not always feasible in these critically ill patients who can be coagulopathic. Furthermore, microbiological yield of the fungus in respiratory specimens can be low [6] and may delay the initiation of definitive treatment.

To overcome these challenges, diagnostic-driven strategies using serological biomarkers, in particular, the *Aspergillus* galactomannan (GM) antigen, have been developed to aid the timely diagnosis of IA and guide management decisions including the initiation of therapy [7]. Serial surveillance of serum GM has been a strategy to trigger antifungal treatment and has been shown to reduce the prescription of empiric antifungal therapy [8,9,10,11,12]. Maertens and colleagues assayed the GM antigen index daily in 88 high-risk haematological patients. This was used to trigger antifungal treatment upon verification with CT scanning and bronchoscopy with bronchoalveolar lavage [9]. Prescription of empirical therapy was reduced from 35% to 7.7% in the cohort subjected to routine GM surveillance. This design, replicated in 27 episodes using twice-weekly serial GM immunoassays, resulted in an 11% reduction in empirical therapy use [10]. In one randomised controlled trial (RCT), engaging GM immunoassay coupled with polymerase chain reaction (PCR) to direct treatment plans resulted in 15% of the cohort (*n* = 118) being prescribed empirical antifungals. The corresponding figure was 32% in those who were not subjected to the additional tests (*n* = 122) [11]. Notably, a French RCT cohort (*n* = 293) saw a 35% reduction in the cost of antifungal therapy, even though the incidence of infection was three times higher in the group utilising biomarkers to guide treatment [12]. Empirical antifungal therapy tends to result in over-treatment [13]. This high cost of antifungal treatment may be averted through biomarker-based surveillance strategy.

The introduction of mould-active posaconazole prophylaxis has effectively reduced the incidence of IA in at-risk patients [14,15]. The use of serum GM immunoassay in conjunction with posaconazole prophylaxis was retrospectively examined in Spanish patients with haematological malignancies undergoing intensive chemotherapy or stem-cell transplantation [16,17]. The low incidence of IA breakthrough (1.9%) and seemingly lower positive predictive value of biweekly GM surveillance prompted a revision of recommendations by the Infectious Diseases Society of America (IDSA) [18]. In its updated 2016 clinical guidelines, routine GM immunoassay was recommended only in patients given non-mould-active prophylaxis, but not those prescribed mould-active prophylaxis [19]. The argument against routine GM immunoassay as a means of surveillance was made based on reduced sensitivity of the test in this population. Resources in healthcare are almost always meagre relative to demand; therefore, we ought to incorporate an economic evaluation to inform clinical decisions. In this study, we evaluated the cost-effectiveness of GM-based pre-emptive treatment algorithms in the era of effective anti-mould prophylaxis. Routine GM surveillance with mould-active prophylaxis (represented by posaconazole) was compared against its use with non-mould-active prophylaxis (represented by fluconazole) to rationalise the continuity of biomarker-based surveillance strategy.

## 2. Methods

We assessed the cost-effectiveness of twice-weekly GM surveillance using an economic model and followed the Consolidated Health Economic Evaluation Reporting Standards (CHEERS) to present the analysis [20]. 

### 2.1. Model Structure

**Figure 1:**
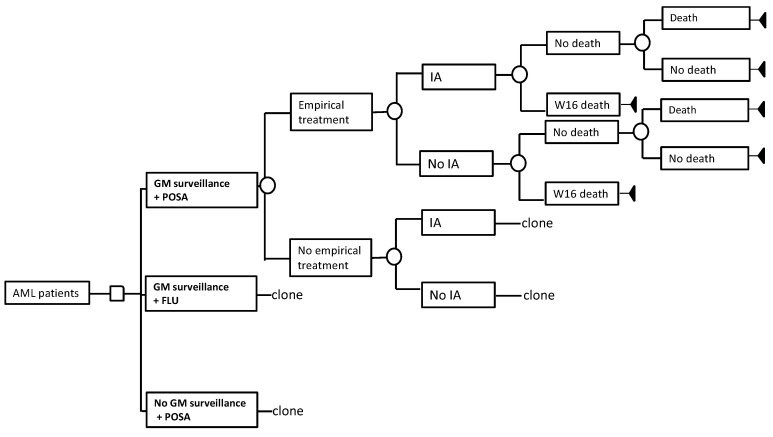
Schematic representation of the decision–analytic model. The model simulated patients with myelosuppression who underwent either twice-weekly routine galactomannan surveillance while receiving antifungal prophylaxis (non-mould-active prophylaxis, i.e., fluconazole, or mould-active prophylaxis, i.e., posaconazole). Patients surviving the IA and the initial 16 weeks (W16) proceeded on to a yearly Markov cycle until death occurred. AML: acute myeloid leukaemia, FLU: fluconazole, GM: galactomannan, IA: invasive aspergillosis, POSA: posaconazole.

We designed a decision–analytic Markov model using TreeAge Pro SuiteTM software 2018 (TreeAge Software Inc., Williamstown, MA, USA) (Figure 1).

The base case consisted of two hypothetical cohorts, 40- and 60-year-old acute myeloid leukaemia (AML) patients reflective of the demographics of the condition [21,22]. These patients entered the model with myelosuppression after undergoing intensive combination induction chemotherapy and were at risk of developing an IA. While receiving antifungal prophylaxis (either non-mould-active prophylaxis represented by fluconazole or mould-active prophylaxis represented by posaconazole), the patients were subjected to either twice-weekly GM surveillance or no biomarker monitoring. When the GM antigen index was positive (i.e., >0.5 optical density index), patients underwent a panel of investigations before the commencement of antifungal therapy. This included full blood count, serum electrolytes and creatinine, liver function tests, chest X-ray, CT scanning of the thorax, blood culture for bacterial and fungi, urinalysis, and urine culture. Patients who despite having negative GM results but were not devoid of clinically concerning IA were subjected to bronchoalveolar lavage fluid assays for bacterial and fungal cultures. In the comparative strategy (i.e., no routine GM surveillance), serum GM immunoassay was performed only upon clinical suspicions for IA on top of the investigations listed above.

Antifungal therapy with voriconazole was instituted for 12 weeks as treatment for IA, as defined by the European Organization for Research and Treatment of Cancer/Mycoses Study Group (EORTC/MSG) [5]. Empiric antifungal treatment was prescribed when patients had persistent neutropenic fevers that failed to defervesce, despite broad-spectrum antibacterial therapy. After 16 weeks, patients who survived the infection and underlying malignancy progressed to the next stage, which projected the risk of death from the underlying disease independently of their history of infections. The model was run according to the screening cycle, i.e., weekly for the initial 16 weeks, followed by a yearly cycle for the lifetime. Clinical outcomes and costs were discounted at 3% annually [23].

### 2.2. Model Inputs

Transition probabilities between health states were derived from published studies and the clinical databases of a 1000-bed national and regional transplant centre in Singapore, where this study was conducted (Table 1). No consent was obtained because this was secondary research without the use of any identifiable data. Sensitivity and specificity of the GM antigen assay at an optical density index cut-off value of 0.5 were set as 82% and 81%, respectively [24]. That is to say, if the disease prevalence was 9%, two patients who have the disease would be missed (sensitivity 82%, 18% false negatives), and 17 patients who do not have the disease would be wrongly labelled (specificity 81%, 19% false negatives). The risk of developing IA was related to the type of prophylaxis [14]. The incidence of IA following effective mould-active prophylaxis would be much lower [14,15,25]. The propensity to initiate empirical antifungal therapy differed based on whether GM surveillance was carried out and the type of prophylaxis [11]. The rate of all-cause death (within 16 weeks of induction chemotherapy) and death from underlying illness were derived from published studies [26,27] and local epidemiological data [28]. We assumed that the likelihood of IA [29] and mortality [25] were similar across the age range. In our local AML cohort, the 5-year overall survival rates were 47.1% and 30.4% in patients aged 40 to 60 years and older than 60 years, respectively [unpublished]. Together with the baseline probabilities of all-cause mortality of the Singaporean population, the five-year overall survival rates for AML undergoing chemotherapy were adjusted as the cohort aged over the time horizon of the analysis.

### 2.3. Resource Use and Cost

The analysis was conducted from a societal perspective, encompassing direct and indirect costs. Direct medical costs were derived from charges in the Singapore public healthcare institution. These included drugs, laboratory and radiological investigations, hospitalisation, and physician consultation fees. We assumed a concurrent 3-week course of antifungal prophylaxis (fluconazole or posaconazole) when GM surveillance was conducted for 4 weeks with two GM tests per week. This has been our local practice. The treatment of IA assumed a 12-week course of voriconazole, weekly investigations, two weeks of hospitalisation, and outpatient clinic visits thereafter. Routine investigations for those receiving IA treatment were full blood count, serum electrolytes, and additional tests such as serum drug levels for voriconazole. Indirect cost included productivity loss in the 40-year-old cohort which was represented by the median wage of the local workforce [29]. Costs were calculated in Singapore dollars (SGD) (SGD 1.36 = USD 1 in November 2020, Monetary Authority of Singapore).

### 2.4. Outcomes

The outcomes of interest were lifetime costs and quality-adjusted life-years (QALYs) gained, which incorporated both the quality (i.e., utility values) and the quantity of life lived. Utility values describe the quality of life (QoL) for different health states according to individuals’ preferences and range from 0 (death) to 1 (perfect health). The additional cost associated with each successful outcome was calculated and presented as the incremental cost-effectiveness ratio (ICER). The strategy was considered as being cost-effective if its ICER was contained within the pre-defined willingness-to-pay threshold of SGD 82,000 (USD 61,000), which was the value of one gross domestic product per capita for Singapore in 2020 [31].

### 2.5. Scenario Analysis

We considered the possibility that mould-active prophylaxis might not be implemented in other healthcare institutions, and thus evaluated the cost-effectiveness of non-mould-active prophylaxis with and without routine GM surveillance. 

### 2.6. Sensitivity Analysis

We used deterministic sensitivity analyses to examine the effect of varying the model inputs on the ICERs. The model inputs were varied one at a time across a plausible range of values. This helped to identify which model inputs were the key drivers of the results. 

Given that the test performance of serum GM immunoassay could be affected by concurrent mould-active antifungal agents [32,33], we evaluated its impact over a range of sensitivity from 80% to 30%.

We used probabilistic sensitivity analysis to account for the uncertainties of all model inputs. The best fitting distributions based on the nature of the data were determined for each model input parameter and sampled through 1000 Monte Carlo iterations. We assigned beta distributions for probabilities and utility scores, and gamma distributions for costs. Results of the probabilistic sensitivity analysis were presented as a cost-effectiveness acceptability curve, indicating the likelihood of GM surveillance being cost-effective over a range of willingness-to-pay threshold values.

## 3. Results

### 3.1. Base Case Analysis

Base case analysis was performed in two categories of patients, at 40 and 60 years old, to match the incidence of AML onset [21,22]. Applying routine GM surveillance in 40-year-old patients receiving prophylaxis with the mould-active agent (posaconazole) was associated with the lowest cost at SGD 11,227 (USD 8255) and greatest benefit in terms of accrued QALYs (5.3272 gained; Table 2). GM surveillance when employed in conjunction with the non-mould-active agent (fluconazole) compounded a higher cost (SGD 12,225 or USD 8989) and accumulated fewer QALYs compared to its use with mould-active prophylaxis. That is to say, routine GM surveillance during mould-active prophylaxis was dominant over the strategy of GM surveillance in patients receiving non-mould-active prophylaxis. A dominant strategy was less costly, although more effective relative to its comparator. Conversely mould-active prophylaxis without GM surveillance was associated with the highest cost (SGD 14,437 or USD 10,615) and accrued 5.3266 QALYs. Overall, in patients receiving mould-active prophylaxis, use of routine GM surveillance was dominant over the strategy of no GM monitoring. In 60-year-old patients receiving prophylaxis with mould-active agent, the total cost accrued was the lowest (SGD 9234 or USD 6790) along with 1.1693 QALYs gained (Table 2). Mould-active prophylaxis without GM surveillance compounded a cost of SGD 12,292 (USD 9039) and 1.1691 QALYs gained. Patients under GM surveillance during non-mould-active prophylaxis accumulated fewer QALYs (1.1597) compared to being given mould-active prophylaxis, along with a cost of SGD 10,132 (USD 7450). In older patients, routine GM surveillance during mould-active prophylaxis remained the dominant strategy (Table 3).

### 3.2. Scenario Analysis

We simulated a scenario where mould-active prophylaxis had not been implemented. Applying routine GM surveillance in patients receiving prophylaxis with the non-mould-active agent (fluconazole) incurred a cost of SGD 12,225 (USD 8989) and 5.3053 QALYs gained (Table S1). Non-mould-active prophylaxis without GM surveillance was associated with the lower QALYs gained (5.3029) but compounded a higher cost (SGD 15,294 or USD 11,246) than the application of routine GM surveillance. Thus, routine GM surveillance was dominant over the strategy of no GM monitoring among patients receiving non-mould-active prophylaxis.

### 3.3. Deterministic Sensitivity Analysis

**Figure 2:**
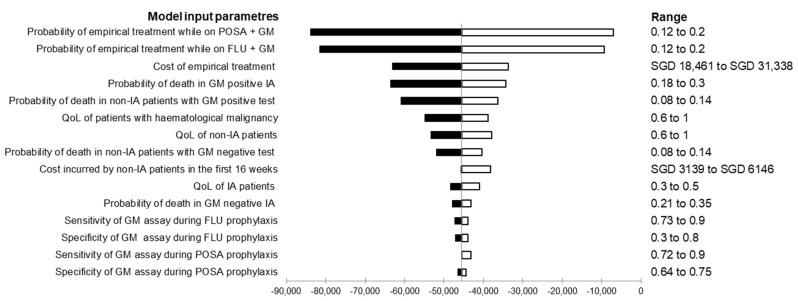
Tornado diagram representing deterministic sensitivity analysis of routine galactomannan surveillance in patients receiving non-mould-active prophylaxis compared to mould-active prophylaxis. Each horizontal bar in the tornado diagram represented the incremental cost-effectiveness ratio (ICER) generated from a range of values evaluated for each parameter. The vertical line represents ICER determined from the base-case analysis. FLU: fluconazole, GM: galactomannan, IA: invasive aspergillosis, IFI: invasive fungal infection, POSA: posaconazole, QoL: quality of life.

Deterministic sensitivity analysis showed that the key factors to influencing the ICER were likelihood of empirical antifungal prescription and the associated cost, represented by the top bars in the tornado diagram (Figure 2). The sensitivity of serum GM immunoassay could be reduced with mould-active prophylaxis [32,33]. To further address how this might affect its cost-effectiveness, we varied the sensitivity from 80% to 30% using one-way sensitivity analysis (Table 4). The cost-effectiveness outcome remained largely unchanged. Even at a lower test sensitivity of 30%, routine GM surveillance during mould-active prophylaxis tended to accrue lower cost and higher QALY over the strategy of no GM monitoring.

### 3.4. Probabilistic Sensitivity Analysis

**Figure 3:**
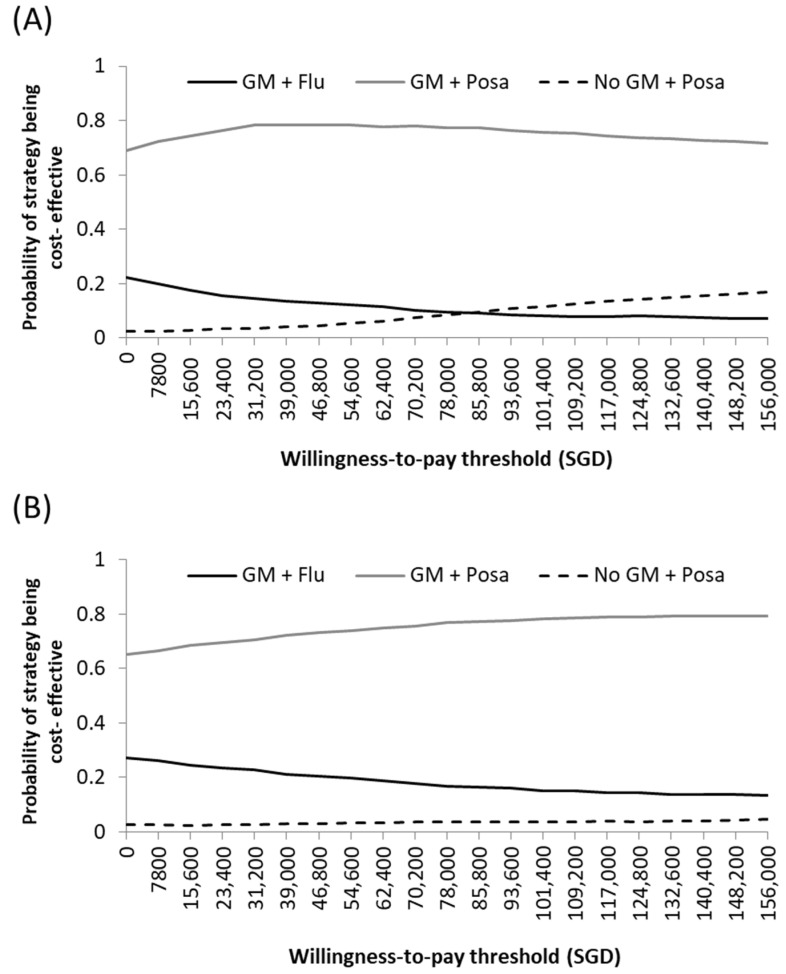
Cost-effectiveness acceptability curve representing probability sensitivity analysis of various prophylaxis-biomarker strategies in (**A**) 40-year-old and (**B**) 60-year-old cohorts. The cost-effectiveness acceptability curve indicated the probability of each strategy being cost-effective over a range of willingness-to-pay (WTP) threshold. The likelihood that the WTP fell within SGD 82,000 per quality-adjusted life-year (QALY) gained (equivalent to one gross domestic product per capita in Singapore, 2020) was 77% when applying routine galactomannan surveillance in conjunction with mould-active prophylaxis across both age ranges. When applying non-mould-active prophylaxis with galactomannan surveillance and mould-active prophylaxis with no galactomannan monitoring, this probability was (**A**) 9% and 10%, respectively, in the 40-year-old cohort, and (**B**) 16% and 4%, respectively, in the 60-year-old cohort. FLU: fluconazole, GM: galactomannan, POSA: posaconazole.

The results of probabilistic sensitivity analysis were presented in the cost-effectiveness acceptability curve. At a willingness-to-pay threshold of SGD 82,000 or USD 61,000 per QALY gained (equivalent to one gross domestic product per capita for Singapore in 2020), the likelihood of GM surveillance being cost-effective while receiving mould-active and non-mould-active antifungal prophylaxis were 77% and 9%, respectively (Figure 3A). The likelihood of no GM monitoring during mould-active prophylaxis being cost-effective was 10%. In 60-year-old patients, the likelihood of GM surveillance during mould-active prophylaxis being cost-effective was unchanged (Figure 3B).

## 4. Discussion

The use of a serum GM immunoassay to guide treatment decision led to a reduction in antifungal prescription. Lower IA breakthrough rates following effective mould-active prophylaxis [14,15,25,34] and assay performance had challenged the practice of serial GM monitoring with the latest IDSA recommendation [19]. Our analysis revealed that it remained cost-effective to continue the practice of routine GM surveillance in patients receiving mould-active prophylaxis. The likelihood of empirical antifungal prescription and drug cost were the most important drivers in determining this cost-effectiveness study. Upon examination in sensitivity analyses, the sensitivity of serum GM immunoassay in the presence of mould-active antifungal agent was not a determinant of its cost-effectiveness.

To date, analysis of the cost-effectiveness analysis of adopting a biomarker-based diagnostic strategy for IA had been restricted to two studies [35,36]. A cost-effectiveness analysis was performed using individual patient cost data from a clinical trial in Australia [35]. The comparison was made, using regular GM and/or *Aspergillus* polymerase chain reaction (PCR) assay in conjunction with CT scan of the thorax, against a standard culture–histology diagnostic approach. This involved twice-weekly serum GM and PCR tests for hospitalised patients and once-weekly tests for outpatients over 26 weeks. The findings from 137 patients found no difference in survival benefit from the intervention. The all-cause mortality was 10.1% (7/69) versus 14.7% (10/68) in the biomarker and standard diagnostic approach, respectively (*p* = 0.573). Extrapolating this observation in their economic model setting, a biomarker diagnostic approach, while not immediately apparent, was deemed to be cost-effective over time with a respectably reduced cost per year of USD 3670 at 5 years [35].

In the United Kingdom, comparisons were made between empirical treatment (initiated in persistent neutropenic fevers which failed to respond after 72 to 96 h of treatment with broad-spectrum antibiotics) and a biomarker diagnostic approach where treatment was driven by positive GM and/or *Aspergillus* PCR findings [36]. The model input parameters were drawn from published studies and expert opinions. A biomarker diagnostic approach led to a reduction of 32% in total hospitalisation cost and 41% reduction in antifungal usage. This biomarker diagnostic approach was cost-effective based on a 10% invasive fungal infection incidence in patients with haematological malignancies undergoing chemotherapy or haematopoietic stem cell transplantation. Similar to our analysis, but with a higher upfront cost of GM surveillance, the overall cost incurred by patients with GM surveillance was lower, indicating that this was a cost-effective strategy. The use of GM and PCR incurred an additional GBP 27 (USD 34) in the United Kingdom, which was a small fraction as compared to our model (USD 518).

In contrast to previous pharmacoeconomic models, we incorporated various bed-side elements of prophylaxis-biomarker strategy. We attempted to streamline subsequent investigations in response to the GM antigen index. We also applied the rate of IA breakthrough as determined by the type of prophylaxis. This helped to establish if it was justified to withdraw the test given the proven effectiveness of mould-active agents [14,15]. To mimic real-world clinical decisions, we varied the propensity to initiate empirical antifungal treatment stratified by the prophylaxis-biomarker strategy [11]. During mould-active prophylaxis, the likelihood of receiving empirical treatment decreased by 50% with GM surveillance. With that, savings of USD 10,000 from each 12-week course of voriconazole were computed. Our analysis revealed that the use of empirical antifungal treatment was a key driver in the cost-effectiveness of GM surveillance. Clinical studies have shown benefits of routine GM surveillance in terms of reduced cost and the prescription of antifungal treatment, but not in mortality rates [8,10,12]. This was made possible through serial GM surveillance as a trigger to guide further investigation and initiation of antifungal treatment as opposed to empirical prescription applied in prolonged neutropenic fevers unresolved by board-spectrum antibiotics [13].

The sensitivity of serum GM immunoassay was factored into our model [30,37,38]. The GM assay could be affected by the administration of an antifungal agent [39]. Earlier meta-analyses had estimated the sensitivity and specificity of GM in sera to be 82% and 81%, respectively, using an optical density index of 0.5 as the cut-off value [24]. However, more recent reviews had put the sensitivity of serum GM assay between 40 and 70%, depending on whether the patient was neutropenic [40]. The loss of sensitivity can be compounded by patients receiving a mould-active antifungal agent [33]. The finding by Calmette and colleagues was indicative that GM tests could be affected by posaconazole [32]. When posaconazole concentration was below 0.5 mg/L, one in six IA patients tested negative using an optical density index cut-off value of 0.5. When posaconazole concentration was above 0.5 mg/L, all IA patients were tested negative. By progressively lowering the sensitivity of GM assay from 80% to 30%, we showed that GM sensitivity over this range did not affect the cost-effectiveness (Table 3).

Our study had its limitations. We used QALY, which incorporates life expectancy and quality of life as a measure of effectiveness. There was not any significant trend in survival benefits from the use of a biomarker diagnostic approach as with previous studies. Based on a cohort study of haematological patients receiving fluconazole prophylaxis, GM surveillance led to improved clinical success—defined as patients not using antifungal agents 6 weeks after the end of the episode or the day before subsequent cycles of chemotherapy [8]. The model did not include the cost of adverse events or management of the underlying malignancies because these costs were assumed to be similar in all treatment groups. The generalisability of cost-effectiveness study has been a disputed issue due to the resource utilisation and price structure. We noted a disparity in the cost of GM immunoassays, but this did not appear to be a major concern in the deterministic sensitivity analysis.

In summary, routine GM surveillance during mould-active antifungal prophylaxis was cost-effective with due consideration of reduced test sensitivity and lower incidence of IA breakthrough (compared to fluconazole prophylaxis). Such a strategy remained cost-effective for streamlining investigations and targeting the population for antifungal therapy, thereby reducing the economic burden of overtreatment. This information ought to be taken into consideration when adopting best practices as stipulated by clinical guidelines, while bearing in mind the generalisability of cost-effectiveness study.

## 5. Transparency Declaration

L.Y.A.C. is supported by the Clinician Scientist Award (CSA Senior Investigator), Individual Research Grant (IRG), Bedside & Bench (B&B) Grants, Centre Grant, and the Training Fellowship Award from the National Medical Research Council (NMRC), Singapore. L.Y.A.C. also acknowledges the Aspiration Grant & Summit Research Program, Bench to Bedside Grant and Seed Fund from the National University Health System, Infectious Diseases and Synthetic Biology Translational Research Program of National University of Singapore as well as the Synthetic Biology Research & Development Program of the National Research Foundation, Singapore. L.Y.A.C. has received grant support and has been an advisor/consultant for Pfizer and Merck Sharp and Dohme (MSD), Gilead and Astellas.

## Figures and Tables

**Table 1:** Model input parameters.

Parameters	Base-Case	Uncertainty (Range/Distribution)	Source
**Events**			
**Probability of IA**			
Non-mould-active prophylaxis	0.08	0.06–0.10 (beta)	Cornely et al., 2007 [14]
Mould-active prophylaxis	0.02	0.015–0.025 (beta)	Cornely et al., 2007 [14]
**Probability of receiving empirical treatment**			
No GM test while on non-mould-active prophylaxis	0.34	0.26–0.43 (beta)	Morrissey et al., 2013 [11]
No GM test while on mould-active prophylaxis	0.23	0.17–0.29 (beta)	Morrissey et al., 2013 [11]
GM test while on non-mould-active prophylaxis	0.16	0.12–0.20 (beta)	Morrissey et al., 2013 [11]
GM test while on mould-active prophylaxis	0.16	0.12–0.20 (beta)	Morrissey et al., 2013 [11]
**Probability of test being positive** **(sensitivity) in IA patients**			
Non-mould-active prophylaxis	0.82	0.73–0.90 (beta)	Leeflang et al., 2015 [24]
**Probability of test being negative (specificity) in non-IA patients**			
Non-mould-active prophylaxis	0.81	0.72–0.90 (beta)	Leeflang et al., 2015 [24]
**Probability of all-cause death in IA patients**			
No GM test	0.25	0.19–0.31 (beta)	Chai et al., 2012 [30], post hoc analysis
GM test positive	0.28	0.21–0.35 (beta)	Jung et al., 2018 [27]
GM test negative	0.24	0.18–0.30 (beta)	Jung et al., 2018 [27]
**Probability of all-cause death in non-IA patients**	0.11	0.08–0.14 (beta)	local hospital data
**Probabilities of death after 16 weeks**			
First year	age-specific AML cohort		local hospital data (NUH AML Database)
Subsequent year	life table		Department of Statistics, Singapore [28]
**Costs (SGD)**			
**Investigation (GM test positive)**			
Serum GM antigen index	704	528–880 (gamma)	local hospital data
Radiography	547	517–577 (gamma)	local hospital data
Septic work-up	431	431–431 (gamma)	local hospital data
**Investigation (GM test negative)**			
Serum GM antigen index (8 tests)	704	528–880 (gamma)	local hospital data
Radiography	547	517–577 (gamma)	local hospital data
Septic work-up	431	431–431 (gamma)	local hospital data
Additional investigations with bronchoscopy (including GM in BAL)	971	971–971 (gamma)	local hospital data
**Investigation (No routine GM test)**			
Serum GM antigen index on suspicions	196	88–196 (gamma)	local hospital data
Radiography	547	517–577 (gamma)	local hospital data
Septic work-up on suspicions	431	431–431 (gamma)	local hospital data
Additional investigations with bronchoscopy (including GM in BAL)	971	971–971 (gamma)	local hospital data
**Treatment of IA**			
IA infection (12-week course of voriconazole)	14,280	11,900–16,660 (gamma)	local hospital data
Outpatient visits	196	98–256 (gamma)	local hospital data
Productivity loss	2116	1058–4232 (gamma)	local hospital data
**Laboratory investigation (liver, renal panel, and full blood count)**			
IA patients	609	483–736 (gamma)	local hospital data
Non-IA patients	165	143–154 (gamma)	local hospital data
**Hospitalisation**			
IA patients	5992	4494–8988 (gamma)	local hospital data
Non-IA patients	2996	2996–5992 (gamma)	local hospital data

AML: acute myeloid leukaemia, BAL: bronchoalveolar lavage fluid, IA: invasive aspergillosis, GM: galactomannan.

**Table 2:** Total cost, effectiveness, and incremental cost-effectiveness ratio of various prophylaxis-biomarker strategies in the 40-year-old cohort.

Strategy	Total Cost	Incremental Cost	QALYs Gained	Incremental QALY	ICER ^#^
Routine GM assay during mould-active prophylaxis	SGD 11,227 (USD 8255)	-	5.3272	-	-
Routine GM assay during non-mould-active prophylaxis	SGD 12,225 (USD 8989)	SGD 998 (USD 734)	5.3053	−0.0219	dominated
No GM assay during mould-active prophylaxis	SGD 14,437 (USD 10,615)	SGD 3210 (USD 2316)	5.3266	−0.0006	dominated

ICER: incremental cost-effectiveness ratio, GM: galactomannan, QALY: quality-adjusted life-year. ^#^ A dominated strategy is more costly and less effective relative to its comparator.

**Table 3:** Total cost, effectiveness, and incremental cost-effectiveness ratio of various prophylaxis-biomarker strategies in the 60-year-old cohort.

Strategy	Total Cost	Incremental Cost	QALYs Gained	Incremental QALY	ICER ^#^
Routine GM assay during mould-active prophylaxis	SGD 9234 (USD 6790)	-	1.1693	-	-
Routine GM assay during non-mould-active prophylaxis	SGD 10,132 (USD 7450)	SGD 898 (USD 660)	1.1597	−0.0096	dominated
No GM assay during mould-active prophylaxis	SGD 12,292 (USD 9039)	SGD 3058 (USD 2249)	1.1691	−0.0002	dominated

ICER: incremental cost-effectiveness ratio, GM: galactomannan, QALY: quality-adjusted life-year. ^#^ A dominated strategy is more costly and less effective relative to its comparator.

**Table 4:** Total cost, effectiveness, and incremental cost-effectiveness ratio when the sensitivity of serum galactomannan immunoassay decreased in patients receiving mould-active prophylaxis.

Sensitivity of GM Assay	Strategy	Total Cost	Incremental Cost	QALYs Gained	Incremental QALY	ICER ^#^
-	No GM assay during mould-active prophylaxis	SGD 14,437 (USD 10,615)		5.3266	-	-
80%	Routine GM assay during mould-active prophylaxis	SGD 11,227 (USD 8255)	−SGD 3210 (USD 2316)	5.3272	0.0006	dominant
70%	Routine GM assay during mould-active prophylaxis	SGD 11,227 (USD 8255)	−SGD 3206 (USD 2357)	5.3274	0.0007	dominant
60%	Routine GM assay during mould-active prophylaxis	SGD 11,227 (USD 8255)	−SGD 3201 (USD 2354)	5.3275	0.0009	dominant
50%	Routine GM assay during mould-active prophylaxis	SGD 11,227 (USD 8255)	−SGD 3196 (USD 2350)	5.3276	0.0010	dominant
40%	Routine GM assay during mould-active prophylaxis	SGD 11,227 (USD 8255)	−SGD 3192 (USD 2347)	5.3278	0.0012	dominant
30%	Routine GM assay during mould-active prophylaxis	SGD 11,227 (USD 8255)	−SGD 3187 (USD 2343)	5.3279	0.0013	dominant

ICER: incremental cost-effectiveness ratio, GM: galactomannan, QALY: quality-adjusted life-year. ^#^ A dominant strategy was less costly, although more effective relative to its comparator.

## Supplementary Materials

The following are available online at https://www.mdpi.com/article/10.3390/jof7060417/s1, Table S1: Total cost, effectiveness and incremental cost-effectiveness ratio of routine GM surveillance compared to no monitoring in patients receiving non-mould-active prophylaxis.

## References

[1] Kontoyiannis D.P., Marr K.A., Park B.J., Alexander B.D., Anaissie E.J., Walsh T.J., Ito J., Andes D.R., Baddley J.W., Brown J.M. (2010). Prospective surveillance for invasive fungal infections in hematopoietic stem cell transplant recipients, 2001–2006: Overview of the Transplant-Associated Infection Surveillance Network (TRANSNET) Database. Clin. Infect. Dis..

[2] Pagano L., Caira M., Candoni A., Offidani M., Fianchi L., Martino B., Pastore D., Picardi M., Bonini A., Chierichini A. (2006). The epidemiology of fungal infections in patients with hematologic malignancies: The SEIFEM-2004 study. Haematologica.

[3] Pagano L., Caira M., Nosari A., van Lint M.T., Candoni A., Offidani M., Aloisi T., Irrera G., Bonini A., Picardi M. (2007). Fungal infections in recipients of hematopoietic stem cell transplants: Results of the SEIFEM B-2004 study—Sorveglianza Epidemiologica Infezioni Fungine Nelle Emopatie Maligne. Clin. Infect. Dis..

[4] Slobbe L., Polinder S., Doorduijn J.K., Lugtenburg P.J., el Barzouhi A., Steyerberg E.W., Rijnders B.J.A. (2008). Outcome and medical costs of patients with invasive aspergillosis and acute myelogenous leukemia-myelodysplastic syndrome treated with intensive chemotherapy: An observational study. Clin. Infect. Dis..

[5] De Pauw B., Walsh T.J., Donnelly J.P., Stevens D.A., Edwards J.E., Calandra T., Pappas P.G., Maertens J., Lortholary O., Kauffman C.A. (2008). Revised definitions of invasive fungal disease from the European Organization for Research and Treatment of Cancer/Invasive Fungal Infections Cooperative Group and the National Institute of Allergy and Infectious Diseases Mycoses Study Group (EORTC/MSG) Consensus Group. Clin. Infect. Dis..

[6] Shah A.A., Hazen K.C. (2013). Diagnostic accuracy of histopathologic and cytopathologic examination of Aspergillus species. Am. J. Clin. Pathol..

[7] Herbrecht R., Letscher-Bru V., Oprea C., Lioure B., Waller J., Campos F., Villard O., Liu K., Natarajan-Amé S., Lutz P. (2002). Aspergillus galactomannan detection in the diagnosis of invasive aspergillosis in cancer patients. J. Clin. Oncol..

[8] Harricharan S., Biederman K., Bombassaro A.M., Lazo-Langner A., Elsayed S., Fulford A., Delport J.A., Xenocostas A. (2018). Adherence to, and outcomes of, a galactomannan screening protocol in high-risk hematology patients. Curr. Oncol..

[9] Maertens J., Theunissen K., Verhoef G., Verschakelen J., Lagrou K., Verbeken E., Wilmer A., Verhaegen J., Boogaerts M., van Eldere J. (2005). Galactomannan and computed tomography-based preemptive antifungal therapy in neutropenic patients at high risk for invasive fungal infection: A prospective feasibility study. Clin. Infect. Dis..

[10] Tan B.H., Low J.G.H., Chlebicka N.L., Kurup A., Cheah F.K., Lin R.T.P., Goh Y.T., Wong G.C. (2011). Galactomannan-guided preemptive vs. empirical antifungals in the persistently febrile neutropenic patient: A prospective randomized study. Int J. Infect. Dis..

[11] Morrissey C.O., Chen S.C., Sorrell T.C., Milliken S., Bardy P.G., Bradstock K.F., Szer J., Halliday C.L., Gilroy N.M., Moore J. (2013). Galactomannan and PCR versus culture and histology for directing use of antifungal treatment for invasive aspergillosis in high-risk haematology patients: A randomised controlled trial. Lancet Infect. Dis..

[12] Cordonnier C., Pautas C., Maury S., Vekhoff A., Farhat H., Suarez F., Dhédin N., Isnard F., Ades L., Kuhnowski F. (2009). Empirical versus preemptive antifungal therapy for high-risk, febrile, neutropenic patients: A randomized, controlled trial. Clin. Infect. Dis..

[13] Fung M., Kim J., Marty F.M., Schwarzinger M., Koo S. (2015). Meta-Analysis and Cost Comparison of Empirical versus Pre-Emptive Antifungal Strategies in Hematologic Malignancy Patients with High-Risk Febrile Neutropenia. PLoS ONE.

[14] Cornely O.A., Maertens J., Winston D.J., Perfect J., Ullmann A.J., Walsh T.J., Helfgott D., Holowiecki J., Stockelberg D., Goh Y. (2007). Posaconazole vs. fluconazole or itraconazole prophylaxis in patients with neutropenia. N. Engl. J. Med..

[15] Ullmann A.J., Lipton J.H., Vesole D.H., Chandrasekar P., Langston A., Tarantolo S.R., Greinix H., de Azevedo W.M., Reddy V., Boparai N. (2007). Posaconazole or fluconazole for prophylaxis in severe graft-versus-host disease. N. Engl. J. Med..

[16] Cornely O.A. (2014). Galactomannan testing during mold-active prophylaxis. Clin. Infect. Dis..

[17] Duarte R.F., Sánchez-Ortega I., Cuesta I., Arnan M., Patiño B., de Sevilla A.F., Gudiol C., Ayats J., Cuenca-Estrella M. (2014). Serum galactomannan-based early detection of invasive aspergillosis in hematology patients receiving effective antimold prophylaxis. Clin. Infect. Dis..

[18] Walsh T.J., Anaissie E.J., Denning D.W., Herbrecht R., Kontoyiannis D.P., Marr K.A., Morrison V.A., Segal B.H., Steinbach W.J., Stevens D.A. (2008). Treatment of aspergillosis: Clinical practice guidelines of the Infectious Diseases Society of America. Clin. Infect. Dis..

[19] Patterson T.F., Thompson G.R., Denning D.W., Fishman J.A., Hadley S., Herbrecht R., Kontoyiannis D.P., Marr K.A., Morrison V.A., Nguyen M.H. (2016). Practice Guidelines for the Diagnosis and Management of Aspergillosis: 2016 Update by the Infectious Diseases Society of America. Clin. Infect. Dis..

[20] Husereau D., Drummond M., Petrou S., Carswell C., Moher D., Greenberg D., Augustovski F., Briggs A.H., Mauskopf J., Loder E. (2013). Consolidated Health Economic Evaluation Reporting Standards (CHEERS)—Explanation and elaboration: A report of the ISPOR Health Economic Evaluation Publication Guidelines Good Reporting Practices Task Force. Value Health.

[21] Appelbaum F.R., Gundacker H., Head D.R., Slovak M.L., Willman C.L., Godwin J.E., Anderson J.E., Petersdorf S.H. (2006). Age and acute myeloid leukemia. Blood.

[22] Juliusson G., Lazarevic V., Horstedt A.S., Hagberg O., Hoglund M., Swedish Acute Leukemia Registry Group (2012). Acute myeloid leukemia in the real world: Why population-based registries are needed. Blood.

[23] (2018). Drug Evaluation Methods & Process.

[24] Leeflang M.M.G., Debets-Ossenkopp Y.J., Wang J., Visser C.E., Scholten R.J.P.M., Hooft L., Bijlmer H.A., Reitsma J.B., Zhang M., Bossuyt P.M.M. (2015). Galactomannan detection for invasive aspergillosis in immunocompromised patients. Cochrane Database Syst. Rev..

[25] Ananda-Rajah M.R., Grigg A., Downey M.T., Bajel A., Spelman T., Cheng A., Thursky K.T., Vincent J., Slavin M.A. (2012). Comparative clinical effectiveness of prophylactic voriconazole/posaconazole to fluconazole/itraconazole in patients with acute myeloid leukemia/myelodysplastic syndrome undergoing cytotoxic chemotherapy over a 12-year period. Haematologica.

[26] Herbrecht R., Denning D.W., Patterson T.F., Bennett J.E., Greene R.E., Oestmann J., Kern W.V., Marr K.A., Ribaud P., Lortholary O. (2002). Voriconazole versus amphotericin B for primary therapy of invasive aspergillosis. N. Engl. J. Med..

[27] Jung J., Kim M.Y., Chong Y.P., Lee S., Choi S., Kim Y.S., Woo J.H., Kim S. (2018). Clinical characteristics, radiologic findings, risk factors and outcomes of serum galactomannan-negative invasive pulmonary aspergillosis. J. Microbiol. Immunol. Infect..

[28] Complete Life Tables 2015–2016 for Singapore Resident Population Department of Statics S. www.singstat.gov.sg.

[29] Labour Market Statistical Information, Ministry of Manpower 2017, Singapore. http://stats.mom.gov.sg/Pages/Income-Summary-Table.aspx.

[30] Chai L.Y.A., Kullberg B., Johnson E.M., Teerenstra S., Khin L.W., Vonk A.G., Maertens J., Lortholary O., Donnelly P.J., Schlamm H.T. (2012). Early serum galactomannan trend as a predictor of outcome of invasive aspergillosis. J. Clin. Microbiol..

[31] WHO Global Health Expenditure Atlas (2012). World Health Organisation. www.who.int/nha/atlas.pdf.

[32] Calmettes C., Gabriel F., Blanchard E., Servant V., Bouchet S., Kabore N., Forcade E., Leroyer C., Bidet A., Latrabe V. (2018). Breakthrough invasive aspergillosis and diagnostic accuracy of serum galactomannan enzyme immune assay during acute myeloid leukemia induction chemotherapy with posaconazole prophylaxis. Oncotarget.

[33] Marr K.A., Laverdiere M., Gugel A., Leisenring W. (2005). Antifungal therapy decreases sensitivity of the Aspergillus galactomannan enzyme immunoassay. Clin. Infect. Dis..

[34] Vehreschild J.J., Rüping M.J.G.T., Wisplinghoff H., Farowski F., Steinbach A., Sims R., Stollorz A., Kreuzer K., Hallek M., Bangard C. (2010). Clinical effectiveness of posaconazole prophylaxis in patients with acute myelogenous leukaemia (AML): A 6 year experience of the Cologne AML cohort. J. Antimicrob. Chemother..

[35] Macesic N., Morrissey C.O., Liew D., Bohensky M.A., Chen S.C., Gilroy N.M., Milliken S.T., Szer J., Slavin M.A. (2017). Is a biomarker-based diagnostic strategy for invasive aspergillosis cost effective in high-risk haematology patients?. Med. Mycol..

[36] Barnes R., Earnshaw S., Herbrecht R., Morrissey O., Slavin M., Bow E., McDade C., Charbonneau C., Weinstein D., Kantecki M. (2015). Economic Comparison of an Empirical Versus Diagnostic-Driven Strategy for Treating Invasive Fungal Disease in Immunocompromised Patients. Clin. Ther..

[37] Chai L.Y.A., jan Kullberg B., Earnest A., Johnson E.M., Teerenstra S., Vonk A.G., Schlamm H.T., Herbrecht R., Netea M.G., Troke P.F. (2014). Voriconazole or amphotericin B as primary therapy yields distinct early serum galactomannan trends related to outcomes in invasive aspergillosis. PLoS ONE.

[38] Fisher C.E., Stevens A.M., Leisenring W., Pergam S.A., Boeckh M., Hohl T.M. (2013). The serum galactomannan index predicts mortality in hematopoietic stem cell transplant recipients with invasive aspergillosis. Clin. Infect. Dis..

[39] Marr K.A., Balajee S.A., McLaughlin L., Tabouret M., Bentsen C., Walsh T.J. (2004). Detection of galactomannan antigenemia by enzyme immunoassay for the diagnosis of invasive aspergillosis: Variables that affect performance. J. Infect. Dis..

[40] Zhou W., Li H., Zhang Y., Huang M., He Q., Li P., Zhang F., Shi Y., Su X. (2017). Diagnostic Value of Galactomannan Antigen Test in Serum and Bronchoalveolar Lavage Fluid Samples from Patients with Nonneutropenic Invasive Pulmonary Aspergillosis. J. Clin. Microbiol..

