# Supplementary material for: Cost-Effectiveness of Serum Galactomannan Surveillance during Mould-Active Antifungal Prophylaxis
Source: J Fungi (Basel). 2021 May 26;7(6):417. doi: 10.3390/jof7060417 (PMC8227639; doi:10.3390/jof7060417)
Supplement: Supplementary file 1 [file jof-07-00417-s001.zip › jof-1199735-supplementary.pdf]

Table S1. Total cost, effectiveness and incremental cost-effectiveness ratio of routine GM surveillance compared to no monitoring in patients receiving non-mould-active prophylaxis.

Strategy	Total Cost	Incremental Cost	QALY Gained	Incremental QALY	ICER [#]
Routine GM assay during non-mould-active prophylaxis	SGD 12,225 (USD 8989)	-	5.3053	-	
No GM assay during non-mould-active prophylaxis	SGD 15,294 (USD 11,246)	SGD 3069 (USD 2257)	5.3029	−0.0024	dominated

ICER: incremental cost-effectiveness ratio, GM: galactomannan, QALY: quality-adjusted life-year. [#]A dominated strategy is more costly and less effective relative to its comparator.
